# Associations between aortic pulse wave velocity and aortic and carotid vessel wall thickness in patients with hypertension: assessment with MRI

**DOI:** 10.1186/1532-429X-13-S1-P72

**Published:** 2011-02-02

**Authors:** Anne Brandts, Albert de Roos, Saskia G van Elderen, Lucia J Kroft, Stijntje D Roes, Johan H Reiber, Rob J van der Geest, Jos J Westenberg

**Affiliations:** 1 Leiden University Medical Center, Leiden, Netherlands

## Introduction

Hypertension puts continuous strain on arteries, resulting in arterial wall alterations such as vessel wall thickening and vessel wall stiffening. Due to the availability of suitable acoustic windows, vessel wall thickness (VWT) is studied traditionally by ultrasound from intima-media thickness in the carotid arteries. Arterial vessel wall stiffness can be expressed by Pulse Wave Velocity (PWV), the propagation speed of the systolic wave front through the aorta. For studying direct associations between PWV and VWT, it is desired to sample VWT in the aorta.

## Purpose

To evaluate associations between aortic and carotid wall thickness and aortic PWV and also with age in subjects with and without hypertension using a comprehensive MRI-approach.

## Methods

Fifteen patients (10 women, mean age 49±14years) with hypertension defined as systolic blood pressure>140mmHg and/or diastolic blood pressure>90mmHg before antihypertensive medication was instituted, were included. Fifteen age- and gender-matched healthy volunteers without history of cardiovascular disease were included for comparison.

PWV-assessment was performed on 1.5T MRI-scanner (Philips Medical Systems, Best, the Netherlands) according to transit-time method by applying velocity-encoded MRI with high temporal resolution at the aortic arch. At the same day, aortic and carotid vessel wall imaging was performed on 3T MRI-scanner (Philips Medical Systems, Best, the Netherlands) using three-dimensional (for aorta) and multi-two-dimensional (for carotid artery) dual-inversion black-blood gradient-echo sequences with 0.5×0.5mm^2^ in-plane resolution, spectral-selective fat suppression and navigator respiratory-compensation (for aorta). Ten slices (aorta) and eight slices (common carotid artery) were analyzed using in-house developed software VesselMass. Luminal and outer wall boundaries were manually segmented. Mean VWT was computed and indexed for Body Surface Area (BSA):

∑ [(area vessel wall × slice thickness) / total number of slices]/BSA.

## Results

**Figure 1:**
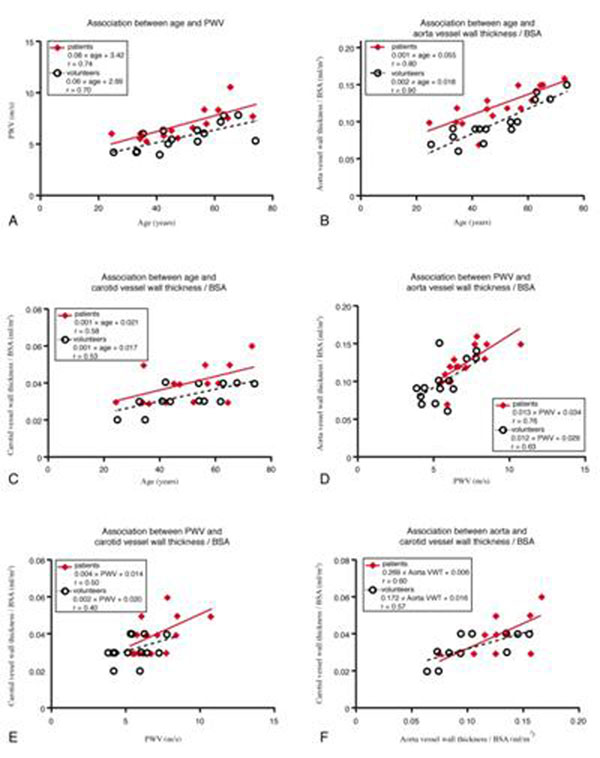
Associations between aortic PWV and aortic and carotid VWT indexed for BSA, and with age.

Hypertensive patients presented with higher PWV and aortic and carotid VWT than healthy volunteers (Table 1). Associations between PWV, aortic and carotid VWT and with age are presented in Figure 1. PWV and aortic and carotid VWT were all significantly associated with age, and all associations were not different between patients and volunteers. Association between aortic PWV and aortic VWT was stronger than for aortic PWV and carotid VWT. Finally, aortic VWT correlated with carotid VWT.

**Table 1:** MRI results

	Hypertension	Volunteers	p-value t-test
Aortic PWV (m/s)	7.0 ± 1.4	5.7 ± 1.3	0.011
Aortic wall thickness/BSA	0.12 ± 0.03	0.10 ± 0.03	<0.001
Carotid wall thickness/BSA	0.04 ± 0.01	0.03 ± 0.01	0.014

## Conclusions

MRI presents associations between aortic and carotid VWT and aortic PWV, and all parameters are increased in patients with hypertension versus age- and gender-matched healthy volunteers.

